# Pharmacokinetic and Bioavailability Studies of Embelin after Intravenous and Oral Administration to Rats

**DOI:** 10.1155/2019/9682495

**Published:** 2019-03-20

**Authors:** Zhen Li, Shu-jing Chen, Xie-an Yu, Jin Li, Xiu-mei Gao, Jun He, Yan-xu Chang

**Affiliations:** ^1^Tianjin State Key Laboratory of Modern Chinese Medicine, Tianjin University of Traditional Chinese Medicine, Tianjin, 300193, China; ^2^Tianjin Key Laboratory of Phytochemistry and Pharmaceutical Analysis, Tianjin University of Traditional Chinese Medicine, Tianjin, 300193, China

## Abstract

Embelin exhibits the broad bioactivities such as antitumor, antifertility, antidiabetic, anti-inflammatory, antioxidant, anticonvulsant, anxiolytic, antimicrobial, and hepatoprotective activity. In order to further understand the pharmacokinetic characteristics and oral bioavailability of embelin* in vivo*, the concentration of embelin in rat plasma was determined by a sensitive high-performance liquid chromatography with diode array detector (HPLC-DAD). The preparation of samples was accomplished by a simple precipitating protein with methanol. Emodin was selected as the internal standard (IS). Embelin and IS were completely separated on an analytical column (Extend-C_18_, 4.6 × 250 mm, 5 *μ*m) using 0.1% phosphoric acid in methanol and 0.1% phosphoric acid in aqueous solution (90:10,* v/v*) as the mobile phase. The lower limit of quantification was 0.15 *μ*g/mL. Oral bioavailability of embelin was 30.2 ± 11.9%. This study could provide the information about pharmacokinetics and oral bioavailability of embelin, which was useful to assess the clinic efficacy and safety and promote further development of embelin.

## 1. Introduction

**Figure 1:**
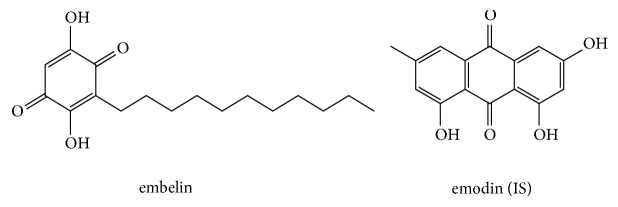
Chemical structures of embelin and emodin (IS).

*Embelia ribes *Burm. f., as an important medicinal plant, has been used extensively in the treatment of various diseases over a long period of time [1, 2]. It belongs to the family Myrsinaceae [3] and is commonly referred to as Vidang or Baibirang [4]. Embelin (2,5-dihydroxy- 3-undecyl-p-benzoquinone, Figure 1) was one of active constituents of* Embelia ribes* Burm. f. [5]. Embelin has been reported to possess many pharmacological effects including antifertility [6], analgesic, anti-inflammatory [7], antioxidant, antidiabetic [8, 9], hepatoprotective [10], anticonvulsant [11], anxiolytic [12], and antimicrobial activity [13]. In addition, the antitumor activity was also reported and numerous articles owned this effect to its ability to enhance TRAIL-induced apoptosis [14], modulate NF-*κ*B signaling pathway for enhancing tumor cell apoptosis [15, 16], and suppress STAT3 [17] as well as Akt/mTOR/S6K1 signaling cascades [18]. The toxicity studies indicated that embelin was safe at doses of 10 mg to 3 g/kg given orally to rats and mice. Notably, a short-term toxicity in female rats, including kidney tubular damage, adrenal hypertrophy, and disintegration of hepatocytes in the liver, was observed after oral administration of embelin for 6 weeks at a dose of 120 mg/kg [19, 20]. Evaluation of safety and efficacy and provision of dose-design information are performed by pharmacokinetics and bioavailability [21]. Therefore, studies on pharmacokinetic behaviors and bioavailability of embelin are important.

Previous studies also became interested in determination of embelin in traditional Chinese medicines, such as the simultaneous determination multiple compounds including embelin or quantitative determination of embelin under the optimum extraction conditions by HPLC [22, 23] and HPTLC [24]. Based on the above considerations, it was a necessary task to carry out the current research for further studying the pharmacokinetics and oral bioavailability of embelin* in vivo*.

Therefore, in the present study, an optimal high-performance liquid chromatographic (HPLC) method with diode array detector (DAD) using emodin as an internal standard (IS) was developed. This validated method was successfully applied to evaluate the pharmacokinetic characteristics and oral bioavailability of embelin following oral and intravenous administration of pure embelin.

## 2. Materials and Methods

### 2.1. Chemicals and Reagents

Standard reference embelin (purity ≥ 98%) and emodin (purity: 99.8%) (Figure 1) were purchased from research technology Co. Ltd., Shanghai research Biotechnology Co., LTD (Shanghai, China), and Chengdu Dest Biotechnology Co. Ltd. (Chengdu, China), respectively. Methanol (Tianjin Concord Science Co. Ltd., Tianjin, China) was of HPLC grade. Phosphoric acid of analytical level was obtained from Tianjin chemical reagent supply and marketing company. Deionized water was purified using a Milli-Q Academic ultra-pure water system (Millipore, Milford, MA, USA).

### 2.2. Apparatus and Chromatographic Conditions

The HPLC analysis was performed on an Agilent 1100 series (Agilent Technologies, USA) consisting of a quaternary pump, a degasser, an autosampler, a column thermostat, and a DAD detector. Chromatographic separation was employed on an analytical column (Extend-C_18_, 4.6 × 250 mm, 5 *μ*m) maintained at 30°C. 0.1% phosphoric acid in methanol-0.1% phosphoric acid in aqueous solution was used as mobile phase. An isocratic elution (90:10,* v/v*) was employed at a flow rate of 1mL/min in a short run time of 12 minutes. The detection wavelength was 280 nm and injection volume was 30 *μ*L.

### 2.3. Preparation of the Stock Solutions and Working Solutions

The primary stock solution of embelin was prepared in methanol at a concentration of 0.5 mg/mL. The stock solution of emodin (IS) was prepared by dissolving the pure emodin in methanol to a concentration of 1 mg/mL and then diluted to a final concentration of 50 *μ*g/mL. Working solutions concluding the calibration standard solutions and quality control (QC) solutions were prepared by further diluting the primary stock solutions. All solutions were stored at 4°C.

The calibration standards (0.15, 0.4, 1, 2, 5, 10, 25, and 50 *μ*g/mL) were freshly prepared by spiking 10 *μ*L of calibration standard solutions in 70 *μ*L of blank plasma. QC samples were prepared at four concentrations (0.4, 1.2, 4, and 40 *μ*g/mL).

### 2.4. Preparation of Samples

To a 70 *μ*L plasma sample, 10 *μ*L IS and 20 *μ*L formic acid were successively added. Thereafter, 400 *μ*L methanol was added to precipitate protein, followed by vortex-mixing for 3 min and centrifuging for 10 min at 14000 rpm. Subsequently, the supernatant was transferred into a new centrifuge tube and evaporated to dryness by nitrogen gas. The dried residue was reconstituted in 70 *μ*L methanol (containing 0.1% phosphoric acid) and then vortexed for 3 min. After a 3 min ultrasound, the mixture was centrifuged for 10 min at 14000 rpm. Finally, 30 *μ*L of the solution was used for analysis.

### 2.5. Method Validation

The specificity was tested by comparing the chromatograms of blank plasma sample (six different batches rats) with the corresponding spiked plasma sample and plasma sample obtained from rat after administration of embelin. The calibration curve was constructed at eight concentration levels and the correlation coefficient (r) was used to evaluate the linearity. The lower limit of quantification (LLOQ) defined as the lowest concentration in the calibration curve was evaluated by the signal to noise ratio (S/N ≥ 5). The intraday precision and accuracy were estimated by analyzing six replicate QC samples at four concentrations in one day. The interday precision and accuracy was carried out once a day for 3 continuous days. The recovery was determined at four concentrations and evaluated by comparing the peak area in processed plasma samples with that in nonprocessed samples. The stability of embelin in plasma containing autosampler 24 h stability and the freeze/thaw cycle stability for three times and the long-term stability for one month were assessed by analyzing QC samples (n = 6) at four concentrations which were stored at the autosampler, -20°C and -80°C, respectively.

### 2.6. Pharmacokinetic Studies

Male Sprague Dawley rats (260-300 g) were bred under an environmentally controlled laboratory with standard food and water and allowed to acclimatize for a week prior to experiments. The rats were fasted for 12 h and allowed free access to water before administration. Twelve rats were randomly divided into two equal groups. The first group was given embelin at an oral dose of 15 mg/kg. 150*μ*L blood samples were collected from the suborbital vein at 0.083, 0.167, 0.25, 0.5, 0.75, 1, 2, 4, 6, 8, 12, and 24 h. The other group was given intravenously at a dose of 5 mg/kg, whose blood samples were collected at 0.033, 0.083, 0.167, 0.25, 0.5, 0.75, 1, 2, 4, 8, 12, and 24 h. After intravenous administration with disposable sterilized syringes, medical cotton ball was pressured on the wound until bloodless. All blood samples were immediately centrifuged to separate plasma at 7000 rpm for 10 min. The obtained plasma was transferred into clean centrifuge tubes and stored at -20°C for subsequent analysis.

### 2.7. Data Analysis

The DAS software (Drug and Statistics 1.0, Medical College of Wannan, China) was used for calculating the pharmacokinetic parameters including AUC, the maximum drug concentration in plasma (C_max_), elimination half-life (t_1/2_), the time for maximal concentration (T_max_), mean residence time (MRT), and choosing the optimum compartment model. The absolute bioavailability (F) was calculated as the following equation:

(1) $$\begin{array}{c} F=\frac{{AUC}_{\left(oral\right)}\times D_{\left(intravenous\right)}}{{AUC}_{\left(intravenous\right)}\times D_{\left(oral\right)}}\times 100\% \end{array}$$

## 3. Result and Discussion

### 3.1. Optimization of HPLC Conditions

The goals of this study were to evaluate the pharmacokinetics and oral bioavailability of embelin, thus a simple, rapid, and sensitive method was used to determine the concentration of embelin in plasma. HPLC conditions concluding the composition and the proportion of the mobile phase, the detection wavelength, and the flow rates were optimized because they play an important role in achieving the goals. Better peak shape, proper retention time, and better sensitivity in a short run time could be obtained when mobile phase consisted of 0.1% phosphoric acid in methanol and 0.1% phosphoric acid in aqueous solution (90:10, v/v), the flow rate was 1.0 mL/min, and the wavelength was 280 nm.

### 3.2. Optimization of Sample Preparation

Preparation of sample is a critical step for the determination of embelin in rat plasma. Acetonitrile and methanol were selected to precipitate protein. The results showed that protein precipitation with methanol could obtain satisfactory extraction recovery. In addition, in order to enhance the extraction recovery, 20 *μ*L formic acid was added and 70 *μ*L 0.1% phosphoric acid in methanol was used to reconstitute the residue.

### 3.3. Method Validation

#### 3.3.1. Specificity

**Figure 2:**
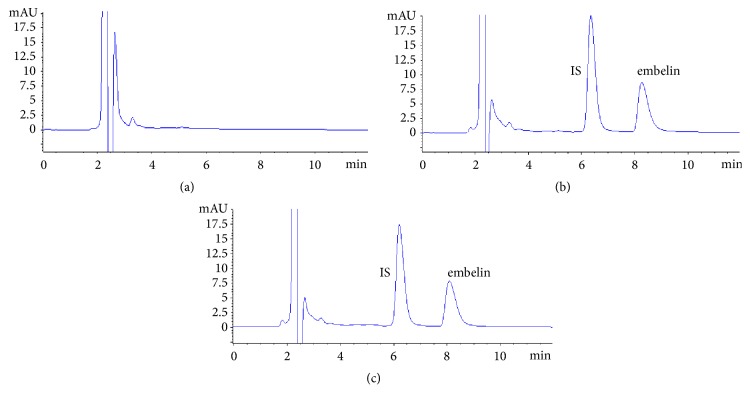
Typical chromatograms of specificity (a) blank rat plasma, (b) spiked blank rat plasma, and (c) plasma sample.

The corresponding representative chromatograms are shown in Figure 2. The retention time of embelin and IS was 8.14 min and 6.36 min, respectively. It was indicated that embelin and IS could be effectively separated. As shown above, no interferences from endogenous substances were observed and no metabolites from embelin were found when real plasma samples were determined.

#### 3.3.2. Linearity and Sensitivity

The calibration curve of embelin was constructed by plotting the peak-area ratios of embelin to IS against the nominal concentrations with a 1/X weight factor. The calibration curve equation of embelin was y = 0.1112x-0.01508 (r = 0.9993), which showed a good linearity over the concentration range of 0.15-50 *μ*g/mL. The calibration curve was applied to determine the concentrations of embelin in plasma samples. The lower limit of quantification was 0.15 *μ*g/mL, which indicated that the developed HPLC method was sensitive.

#### 3.3.3. Precision and Accuracy

The precision was assessed by the relative standard deviation (RSD) and accuracy was determined by comparison of the calculated concentrations using the calibration curve with the nominal concentrations. The results of the intraday and interday precision and accuracy are summarized in Table 1. The RSDs of the intraday and interday were below 15% and accuracy ranged from 85% to 105%. The above results indicated that the method was precise, accurate, and reliable.

#### 3.3.4. Extraction Recovery and Stability

The results of recovery and stability are presented in Tables 1 and 2, respectively. Extraction recoveries of four concentrations levels were higher than 90% and the RSDs of the recovery were within 15%. The data of stability demonstrated that embelin in plasma was stable at the autosampler for 24 h, under the freeze/thaw cycle for three times. The reduction of embelin in plasma stored at -80°C for one month was not obvious, suggesting that embelin in plasma was also stable in a long term.

### 3.4. Pharmacokinetic Studies

#### 3.4.1. Pharmacokinetics of Embelin in Rats after Intravenous Administration

**Figure 3:**
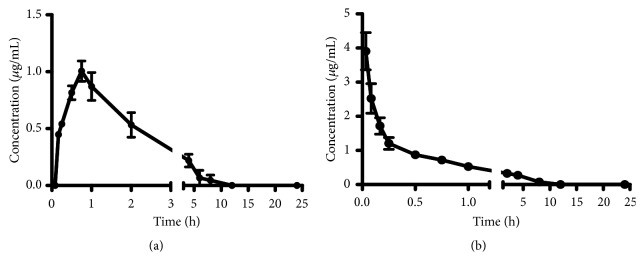
The mean plasma concentration–time profiles of embelin after oral (a) and intravenous (b) administration (n = 6, mean ± SD).

After intravenous administration at a dose of 5 mg/kg, the mean plasma concentration-time profile is illustrated in Figure 3 and the main pharmacokinetic parameters are shown in Table 3. According to DAS analysis, the two-compartment model was most fitted to describe the pharmacokinetics of embelin after intravenous administration. The concentration of embelin in rat plasma distinctly decreased within the first 15 minutes and then slowly decreased. Subsequently, the concentration would be lower than LLOQ during the next 8 h. The elimination was quick [25], which was indicated by the t_1/2_ of 1.52 ± 0.83 h. The value of AUC_(0-24h)_, AUC_(0-*∞*)_, and MRT_(0-24h)_ was 2.17 ± 0.49 *μ*g/mL h, 3.21 ± 0.62 *μ*g/mL h, and 1.28 ± 0.11 h, respectively.

#### 3.4.2. Pharmacokinetics of Embelin in Rats after Oral Administration

The mean plasma concentration-time profile for oral administration at a dose of 15 mg/kg is presented in Figure 3 and the major pharmacokinetic parameters are listed in Table 3. The concentration of embelin in rat plasma could be determined at 10 minutes after an oral administration, which indicated that the absorption of embelin was rapid [26]. In addition, the concentration would also be lower than LLOQ during the next 8 h. According to DAS analysis, the one-compartment model was the most suitable model for oral administration. The value of T_max_ was 0.31 ± 0.18 h, which suggested that embelin can quickly reach the maximum plasma concentration* in vivo*. The t_1/2_ was 1.01 ± 0.58 h, which indicated the elimination was quick, too. The concentration of embelin in plasma was low and the absorption was incomplete, which was indicated by the V/F of more than 6 L and the C_max_ of 1.04 ± 0.21 *μ*g/mL compared with the C_max_ of 3.91 ± 1.34 *μ*g/mL for intravenous administration [27]. This result was related to the inherent poor aqueous solubility of embelin. The value of AUC_(0-tn)_, AUC_(0-*∞*)_, and MRT_(0-tn)_ was 1.97 ± 0.78 *μ*g/mL h_,_ 2.92 ± 0.69 *μ*g/mL h, and 1.49 ± 0.49 h, respectively.

#### 3.4.3. Bioavailability of Embelin in Rats after Administration

Oral bioavailability of embelin was 30.2 ± 11.9%, which showed that oral bioavailability was low. The possible reason was the inherent poor aqueous solubility and the poor absorption property [28].

## 4. Conclusion

An HPLC-DAD method was successfully established and applied to the pharmacokinetic and oral bioavailability studies after oral and intravenous administration of pure embelin. This method was validated in terms of specificity, sensitivity, intraday and intraday precision, recovery, and stability over the linear range. The absorption of embelin* in vivo* was rapid. The oral bioavailability of embelin was 30.2 ± 11.9%. The pharmacokinetic information of embelin* in vivo* is significant for clinical use of embelin.

## Figures and Tables

**Table 1:** Intraday and interday accuracy, precision, and recovery of embelin (n=6).

Concentration (*μ*g mL^−1^)	Intraday		Interday		Recovery	
	Accuracy (%)	RSD (%)	Accuracy (%)	RSD (%)	Accuracy (%)	RSD (%)
0.15	103	2.37	105	2.89	-	-
0.4	97.3	3.83	89.9	7.87	109	10.2
1.2	97.7	4.60	85.4	12.5	107	13.4
4	99.4	7.71	86.2	13.7	94.6	8.42
40	94.4	6.83	97.4	10.9	98.2	8.27

**Table 2:** Stability of embelin (n=6).

Concentration (*μ*g mL^−1^)	Freeze thaw cycles		Autosampler for 24 hours		-20°C for 1 month	
	Accuracy (%)	RSD (%)	Accuracy (%)	RSD (%)	Accuracy (%)	RSD (%)
0.4	96.4	9.87	79.7	4.17	76.8	3.57
1.2	77.9	6.17	82.3	2.72	93.9	2.35
4	103	9.37	84.5	9.27	89.6	5.86
40	116	2.54	118	5.32	103	3.52

**Table 3:** Main pharmacokinetic parameters of embelin (n=6, mean±SD).

Parameters	intravenous (5mg/kg)	oral administration (15mg/kg)
T_max_(h)	0.03±0.00	0.71±0.19
C_max_(*μ*g/mL)	3.91±1.34	1.05±0.21
AUC_(0-24h)_(*μ*g/mL h)	2.17±0.49	1.97±0.78
AUC_(0-*∞*)_(*μ*g/mL h)	3.21±0.62	2.92±0.69
t_1/2_(h)	1.52±0.83	1.01±0.58
MRT_(0-24h)_(h)	1.28±0.11	1.49±0.49
MRT_(0-*∞*)_(h)	3.43±0.41	3.21±1.15

## Data Availability

The data used to support the findings of this study are included within the article.
